# Polymorphic Loci of Adaptively Significant Genes Selection for Determining Nucleotide Polymorphism of *Pinus sylvestris* L. Populations in the Urals

**DOI:** 10.3390/genes15101343

**Published:** 2024-10-21

**Authors:** Nikita Chertov, Yana Sboeva, Yulia Nechaeva, Svetlana Boronnikova, Andrei Zhulanov, Victoria Pechenkina, Ruslan Kalendar

**Affiliations:** 1 Faculty of Biology, Perm State University, Bukireva, 15, 614990 Perm, Russia; syper.gall@mail.ru (N.C.); yana_prishnivskaya@mail.ru (Y.S.); ulia-2012@mail.ru (Y.N.); aumakua.ru@gmail.com (A.Z.); p_viktoria2@mail.ru (V.P.); 2 Perm Agricultural Research Institute—Branch of Perm Federal Research Center Ural Brunch Russian Academy of Sciences, 614532 Perm, Russia; 3 National Laboratory Astana, Nazarbayev University, Astana 010000, Kazakhstan; 4 Institute of Biotechnology HiLIFE, University of Helsinki, 00014 Helsinki, Finland

**Keywords:** adaptive significant genes, locus, nucleotide polymorphism, *Pinus sylvestris* L., the Urals

## Abstract

Background: Scots Pine is one of the main forest-forming species in boreal forests; it has great economic and ecological significance. This study aimed to develop and test primers for detecting nucleotide polymorphisms in genes that are promising for detecting adaptive genetic variability in populations of *Pinus sylvestris* in the Urals and adjacent territories. Objectives: The objects of the study were 13 populations of Scots Pine located in the Perm Territory, Chelyabinsk Region, and the Republic of Bashkortostan. Results: Sixteen pairs of primers to loci of potentially adaptively significant genes were developed, from which three pairs of primers were selected to detect the nucleotide diversity of the studied populations. The indicator of total haplotype diversity determined in the three studied loci varied from 0.620 (*Pinus-12* locus) to 0.737 (*Pinus-11* locus) and, on average, amounted to 0.662. The nucleotide diversity indicators in *P. sylvestris* in the study region were, on average, low (*π* = 0.004, *θ_W_* = 0.013). Their highest values were found at the *Pinus-12* locus (*π* = 0.005; *θ_W_* = 0.032), and the lowest were found at the *Pinus-15* locus (*π* = 0.003; *θ_W_* = 0.002). This indicates that *Pinus-15* is the most conserved of the three studied loci. In the three studied *P. sylvestris* loci associated with adaptation to environmental factors, 97 polymorphic positions were identified. The 13 populations of *P. sylvestris* are characterized by an average level of genetic diversity (*Hd* = 0.662; *π* = 0.004; *θ* = 0.013). Conclusions: The polymorphic loci of adaptively significant genes of *P. sylvestris* can help identify the adaptive potential of pine forests in conditions of increasing ambient temperatures.

## 1. Introduction

High rates of climate change and an increase in the number and duration of droughts leading to large-scale forest fires cause significant damage to forests [1,2,3]. Understanding the genetic basis of population divergence and adaptation is important in population genetics and evolutionary biology. The interaction of evolutionary factors and population structure forms genetic variability and adaptation to changing conditions in woody plant species [4,5,6,7].

Scots Pine (*P. sylvestris* L.), the second most widespread coniferous tree species globally, is highly economically and ecologically important [8]. Forests dominated by the species cover 37% of the total forest area in the world and about 70% of the forest area in the Northern Hemisphere [8,9]. The current range of this species is the result of postglacial recolonization events and the subsequent reduction in pine forests [10,11,12,13]. *P. sylvestris* is a species tolerant of many ecological habitats [14,15]. Scots Pine has high genetic variability, which determines quantitative and qualitative traits, some of which have adaptive value [16,17].

SNPs (single=nucleotide polymorphisms) are most successfully used to characterize genetic diversity at the levels of species and populations. Seventy-eight new SNP marker associations and drought-related traits were found in *Sequoia sempervirens*, and six were found in *Sequoiadenron giganteum* [18]. In addition, exome-derived SNP associations have been identified in several conifer species, such as *Pseudotsuga menziesii* [19] and *Pinus taeda* [20]. They arise due to changes in the nucleotide sequence of DNA when one nucleotide is replaced by another in members of the same species [21,22,23]. Single-nucleotide polymorphisms have been studied in common pine throughout its entire range. At the same time, *P. sylvestris* populations in Russia west of the Ural Mountains differ from other Asian populations [24]. Therefore, to analyze the genetic diversity of *P. sylvestris* in this region, it is necessary to continue selecting polymorphic loci and identifying SNPs in them. In addition, the identification of promising SNPs is relevant for revealing their association with adaptation to drought in a variety of conditions [25,26,27,28].

Thus, the working hypothesis of this study was that the selected polymorphic loci and the SNPs identified in them would allow us to establish and analyze the nucleotide diversity of *P. sylvestris* in the study region and identify possible adaptations of common pine to drought associated with nucleotide polymorphisms in genes potentially associated with drought tolerance.

This study aimed to develop and test primers for identifying nucleotide polymorphisms in genes that are promising for identifying adaptive genetic variability in *P. sylvestris* using populations of this species in the Urals and adjacent territories as an example.

## 2. Materials and Methods

**Figure 1:**
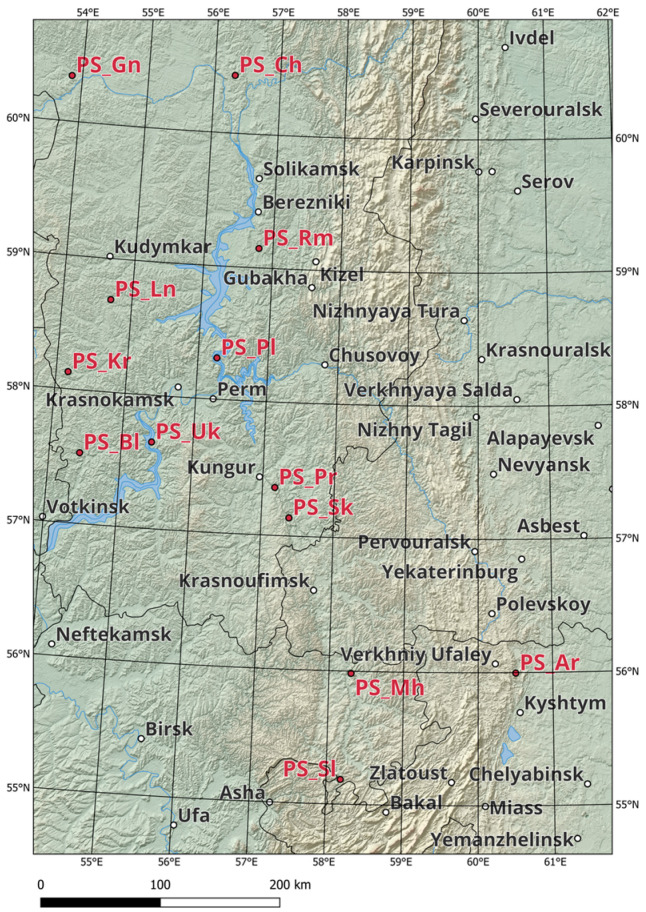
Location diagram of the studied *P. sylvestris* populations. *P. sylvestris* populations in the forests of Perm Krai: *PS_Ch*—Cherdynskoye, *PS_Gn*—Gaynskoye, *PS_Rm*—Bereznikovskoye, *PS_Ln*—Kudymkarskoye, *PS_Pr*—Kishertskoye, *PS_Pl*—Dobryanskoye, *PS_Kr*—Sivinskoye, *PS_Bl*—Tchaikovskoye, *PS_Sk*—Oktyabrskoye, *PS_Uk*—Permskoye; from the Republic of Bashkortostan: *PS_Mh*—Duvanskoye, *PS_Sl*—Salavatskoye; from the Chelyabinsk Region: *PS_Ar*—Kaslinskoye forest.

The objects of the study were 13 populations of *P. sylvestris* L.; 11 were located in the following forests of the Northern and Middle Urals (Perm Krai): Cherdynsky (*PS_Ch*), Gainsky (*PS_Gn*), Bereznikovsky (*PS_Rm*), Kudymkarsky (*PS_Ln*), Dobryansky (*PS_Pl*), Sivinsky (*PS_Kr*), Tchaikovsky (*PS_Bl*), Permsky (*PS_Uk*), Kishertsky (*PS_Pr*), and Oktyabrsky (*PS_Sk*) forestries. Two populations were located in the Republic of Bashkortostan in the Duvansky (*PS_Mh*) and Salavatsky (*PS_Sl*) forests, and one was in the Kaslinsky forest (*PS_Ar*) of the Chelyabinsk region (Figure 1, Table S1). The populations from the Salavatsky forest of the Republic of Bashkortostan (*PS_Sl*) and the Gainsky forest of the Perm region (*PS_Gn*) were at the maximum distance (628 km). The populations from the Kishertsky (*PS_Pr*) and Oktyabrsky (*PS_Sk*) forests of the Perm region were at the minimum distance (28 km).

For molecular genetic analysis, needles were collected separately from 28–31 trees in each population, located no closer than 100–150 m from each other. DNA was isolated using CTAB with further purification using a high-salt gel electroelution trap [29,30] or using an acidic CTAB solution [31].

To analyze nucleotide polymorphism, sixteen pairs of primers to loci of potentially adaptively significant genes were developed (Table 1). The largest databases of single-nucleotide polymorphisms (SNPs) specific to Scots Pine were used to design primers for adaptively significant loci of Scots Pine containing SNPs [32].

*P. sylvestris* does not have GO (gene ontology) identifiers in the SNP database [32]; in addition, the sequences in the database are only 71 nucleotides long; therefore, for all database entries, a search was performed for corresponding contigs in the pine transcriptome taken from the TreeGenes database https://treegenesdb.org/FTP/Transcriptome/TSA/Pisy/Pisy_TSA.fasta, accessed on 18 August 2024). For the identified contigs, automatic functional annotation was performed using InterProScan [33]. From the results (annotations for primer development), 34 contigs with GO identifiers corresponding to adaptively significant genes were selected.

The primers were developed and analyzed using the FastPCR software v.6.9 [34] with the following parameters: the amplified fragment containing SNP length was 400–600 nucleotides, the optimal primer length was 20 nucleotides, and the optimal annealing temperature was 60 °C. *In silico* PCR analysis for primer pairs for the *P. sylvestris* genome was performed using virtualPCR [35].

Pairs of primers that did not form dimers or nonspecific amplification products were selected. A search was performed in the *P. sylvestris* transcriptome used in the previous step to verify the uniqueness of the sequences amplified by these primers. Sequences for which more than one homologous fragment was found were excluded.

After their selection, the most specific primers for the *Pinus-11*, *Pinus-12*, and *Pinus-15* loci were amplified from the *P. sylvestris* total DNA extraction (Table 2).

The purification of PCR products was performed with a mixture of ExoI and FAST-AP enzymes (Thermo Fisher Scientific Inc., Waltham, MA, USA) in a ratio of 0.5:1 at a rate of 1.5 μL of the enzyme mixture per 5 μL of PCR products. According to the program, the reaction was carried out in a GeneAmp PCR System 9700 amplifier (Applied Biosystems, Thermo Fisher Scientific Inc., Waltham, MA, USA): 37 °C—30 min, 80 °C—15 min, cooling to 4 °C.

The BigDye^®^ Terminator v3.1 Cycle Sequencing Kit (Applied Biosystems, USA) was used for sequencing. The forward and reverse sequences from the pair with which the PCR was supplied were used as primers. Amplification was performed in a GeneAmp PCR System 9700 thermal cycler (Applied Biosystems, USA) according to the program: 5 min—94 °C, the next 30 cycles (94 °C—30 s, Ta–45 s, 72 °C—2 min), 72 °C—10 min. The sequencing reaction products were purified from unreacted labeled nucleotides using the BigDye^®^ X Terminator TM Purification Kit (Applied Biosystems, USA).

The study used a method of automated enzymatic sequencing, which included two stages: (1) performing termination reactions (labeling nucleotides with fluorophores) and (2) separating the products of these reactions using capillary electrophoresis. Only the second stage was automated, i.e., separating the labeled DNA fragments, obtaining the fluorophore emission spectrum, and processing the collected data. Capillary electrophoresis of the synthesized sequences was performed in the PCR laboratory of the Department of Botany and Plant Genetics of Perm State National Research University (Russia) on a 24-capillary genetic analyzer, Genetic Analyzer 3500xL (Applied Biosystems, USA), in two directions. On average, three DNA loci nucleotide sequences were sequenced in eight trees from each population. The total length of the nucleotide sequences for 312 trees was 115,128 nucleotides.

Sequenced sequences were edited in the GeneScanv2 program (Applied Biosystems, USA), and the location of polymorphic positions was determined by multiple sequence alignment with the UGENE computer program [36]. Data on the sequenced loci sequences (in the FASTA sequence file format) were compared with those available in the NCBI genetic database using the BLAST 2.2.26+ automatic online alignment system. In the DNASP program [37], the haplotypes were reconstructed, and the following indices of nucleotide polymorphism were calculated on the basis of a comparison of their nucleotide sequences: the number of variable sites (*S*); the number of haplotypes in the population (*h_n_*); the total haplotype diversity (*Hd*) according to M. Nei [38]; the nucleotide diversity (*π*), which estimates the average number of pairwise differences between two DNA sequences and is a measure of the genetic variability of a species or population; and the nucleotide diversity parameter calculated on the basis of the number of mutations (*θ_W_*) or the Watterson estimate [39]. The Watterson estimate is based on the coalescence theory and is calculated from the mutation rate per generation and the number of polymorphic sites [40]. To assess the compliance of the nature of nucleotide substitutions with the neutrality hypothesis, the Tajima D-test was performed for each locus in the studied populations. Its significance was assessed by 10,000 simulations, which allowed us to test the hypothesis of neutrality of the existing polymorphism [41]. Regression analysis was used to analyze the relationship between the level of genetic diversity and various climatic indicators. We obtained 19 basic climatic parameters for each sample from the WorldClim service’s bioclimatic variables database utilizing the raster v3.4–13 package.

## 3. Results and Discussion

We tested 16 primer pairs to loci promising for detecting the genetic diversity of *P. sylvestris* at the population level. As a result of test amplification, nine pairs of primers for the loci *Pinus-1*, *Pinus-2*, *Pinus-5*, *Pinus-6*, *Pinus-7*, *Pinus-10*, *Pinus-11*, *Pinus-12,* and *Pinus-15* showed positive amplification and DNA fragments of the required size (Figure S1). The remaining loci were not amplified or did not reveal amplicons of the required size and, for this reason, were excluded from further study. The main reason for the absence or non-specific amplification may be the incomplete affinity of the primers with the DNA of the studied species. In addition, the synthesis of non-specific fragments can be provoked by excessive or insufficient primer concentration, non-optimal annealing temperature, the concentration of magnesium ions, or the amount of DNA matrix [42]. In this regard, PCR conditions were optimized to obtain high-quality target amplicons. For this purpose, the proportions and concentrations of these components in the PCR mixture and the annealing temperature in several repeated PCRs for each tested locus were varied. As a result, the quality of the PCR products of the studied loci became significantly higher.

For common pine, primers *Pinus-11*, *Pinus-12*, and *Pinus-15* showed successful amplification in PCR of a single fragment and had similar activity according to gene ontology (GO): GO:0009408, response to heat (Table 3). According to GO, heat shock proteins, Annexins, α(B)-crystallins, transcription factors associated with heat stress, and others have this activity.

The search for homologs to the selected loci in the NCBI and UniPlot databases using the BLAST tool revealed that the *Pinus-11* locus is homologous to the small heat shock protein gene (A0AA38G6F5) in the UniPlot database. No homologs were found for this locus in the NCBI database. The *Pinus-12* locus is homologous to the small heat shock protein gene, the α-crystallin domain of the α-crystallin-type small heat shock proteins in the NCBI database (ABK22047). The *Pinus-15* locus is homologous to the *P. taeda* L. transcription factor (the MYB (myeloblastosis) transcription factor) in the NCBI database (AC241314).

The sequenced nucleotide sequences were aligned with each other, and 97 polymorphic positions were found in the sequences of three loci. According to the results of multiple alignments, *Pinus-15* is the most conserved; three polymorphic positions were found in its sequence. The largest number of polymorphic sites was identified in *Pinus-12*–84 substitutions. The largest numbers of haplotypes (*h_n_*) and polymorphic sites (*S*) in *Pinus-11* were found in the *PS_Ar* and *PS_Bl* populations in *Pinus12*–in *PS_Rm*, and *Pinus-15* revealed the largest number of haplotypes and variable sites in the *PS_Ln* population (Table 4).

**Figure 2:**
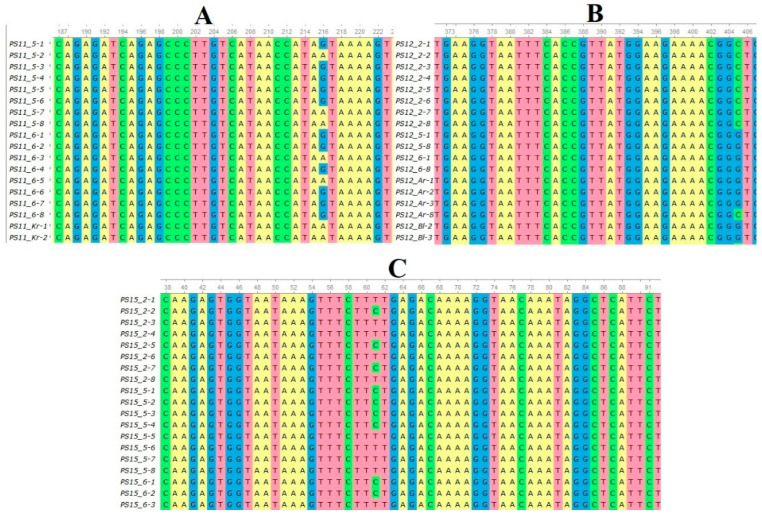
Nucleotide substitutions identified during alignment of sequenced sequences of *P. sylvestris*: (**A**)—in the *Pinus-11* locus; (**B**)—in the *Pinus-12* locus; (**C**)—in the *Pinus-15* locus.

The overall haplotype diversity (*Hd*), a measure of the uniqueness of a particular haplotype in a population, for the three loci studied varied from 0.620 (*Pinus-12* locus) to 0.737 (*Pinus-11* locus), and the indicator was 0.662 on average (Figure 2).

The nucleotide diversity index (*π*), defined as the average number of pairwise nucleotide differences per site between two DNA sequences, is higher at the *Pinus-12* locus (*π*= 0.005) and lower at *Pinus-15* (*π* = 0.003), and the average for the three *P. sylvestris* loci was 0.004 (Table 5).

Watterson’s estimator of nucleotide diversity calculated from the number of mutations (*θ_W_*) also revealed the highest values at the *Pinus-12* locus (*θ_W_* = 0.032) and the lowest at the *Pinus-15* locus (*θ_W_* = 0.002). *Pinus-15* is the most conserved of the studied loci (Table 5).

To test the hypothesis of neutrality of the existing polymorphism, the Tajima D-test (*D_T_*) was used. There are three possible interpretations of the test results: (1) *D_T_* = 0, with π = *θ_W_*, means that the observed values of nucleotide diversity are equal to the expected ones, the population is developing at genetic equilibrium, and there is no expected influence of selection; (2) *D_T_* < 0 with *π < θ_W_* indicates an excess of low-frequency polymorphisms, which may be caused by negative selection or an increase in the population size after its recent reduction—the “bottleneck” effect—and perhaps the adaptability of the gene; (3) *D_T_* > 0 with *π* > *θ_W_* indicates an excess of intermediate-frequency polymorphisms, which may be a result of stabilizing selection or a recent sharp decrease in the population size [41].

It was revealed that for the *Pinus-11* and *Pinus-12* loci, the Watterson estimator values (*θ_W_*) exceeded the nucleotide diversity indices *π*, which indicated an excess of low-frequency alleles and was consistent with the negative values of the Tajima D-test. The closest-to-zero *D_T_* value (−0.890) was found at the *Pinus-11* locus, so it can be assumed that the polymorphism of this locus is selectively neutral. The greatest deviation from the neutral value (*D_T_* = −2.615) was found at the *Pinus-12* locus. It exhibits a positive deviation of the Tajima D-test values (0.925), which indicates an excess of polymorphisms with an intermediate frequency (Table 6).

In general, according to the nucleotide diversity data (Table 6), the most genetically heterogeneous populations are *PS_Ar* (*Hd* = 0.697; π = 0.003; *θ_W_* = 0.003), *PS_Bl* (*Hd* = 0.692; π = 0.003; *θ_W_* = 0.003), and *PS_Rm* (*Hd* = 0.675; π = 0.003; *θ_W_* = 0.03), and the least genetically heterogeneous populations are *PS_Gn* (*Hd* = 0.472; π = 0.005; *θ_W_* = 0.005) and *PS_Pr* (*Hd* = 0.478; π = 0.004; *θ_W_* = 0.004).

According to the regression analysis, there is no direct connection between the level of genetic diversity and various climatic indicators, such as average and maximum temperature and precipitation. However, there is a connection with anthropogenic load. Thus, in the Gainsky forest (*PS_Gn*), the most intensive conifer cutting is carried out, and in the Kasli forest (*PS_Ar*), on the contrary, the intensity of coniferous harvesting is the lowest.

Thus, the nucleotide polymorphism indices of three loci in *P. sylvestris*, common in the Urals and potentially adaptive to environmental factors, were studied, and in those loci, 97 polymorphic positions were identified. The studied populations are characterized by an average level of genetic diversity (*Hd* = 0.662; *π* = 0.004; *θ_W_* = 0.013). A similar level of genetic diversity was found in pine populations in Poland, studied using 32 loci and mitochondrial markers [43,44]. A higher level of haplotype diversity was found in *P. sylvestris* populations from Scotland using 12 loci [45].

## 4. Conclusions

After analyzing genetic databases, 16 primer pairs to loci of adaptively significant genes of *P. sylvestris* were designed and tested in this study, from which three loci, *Pinus-11*, *Pinus-12*, and *Pinus-15*, were selected. Of the loci studied, the *Pinus-12* locus was the most polymorphic. It also showed the greatest deviation from neutrality (*D_T_* = −2.615). The *Pinus-15* locus was the most conserved among the studied loci. It is part of the MYB transcription factor, which is widely involved in plant response to drought. A total of 97 SNPs were detected in the three selected loci.

The selected loci allowed a preliminary analysis of the nucleotide diversity of 13 populations of *P. sylvestris* located in the Urals and adjacent territories based on SNP polymorphism.

The average level of genetic diversity of *P. sylvestris* populations in the study region was revealed (*Hd* = 0.662; *π* = 0.004; *θ_W_* = 0.013). The populations from Kaslinskoye (Hd = 0.697; π = 0.003; *θ_W_* = 0.003), Tchaikovskoye (*Hd* = 0.692; *π* = 0.003; *θ_W_* = 0.003), and Bereznikovskoye forests (*Hd* = 0.675; *π* = 0.003; *θ_W_* = 0.03) showed moderate diversity, and low diversity was observed in the Gainskoye (*Hd* = 0.472; *π* = 0.005; *θ_W_* = 0.005) and Kishertskoye forests (*Hd* = 0.478; *π* = 0.004; *θ_W_* = 0.004) populations.

The studied loci also have a high potential for studying the relationship between polymorphism and the manifestation of adaptability, particularly drought resistance. Since the *Pinus-12* locus has the greatest deviation from neutrality, it is the most promising for further study of drought resistance in *P. sylvestris*. The *Pinus-15* locus is part of the MYB transcription factor, which is widely involved in the plant response to drought [46]. In addition, this transcription factor is involved in regulating the expression of the synthesis of biologically active substances, particularly flavonoids [47]. Studying the polymorphism of this locus in combination with loci associated with drought resistance will contribute to the selection of more productive and tolerant trees of Scots Pine. In connection with climate change, research in this area is very promising and can contribute to increasing the adaptive potential of pine forests in conditions of increasing ambient temperatures.

Different genera of the *Pinaceae* family, including the genus *Pinus*, have an example of SNP associations with drought tolerance [18,19,20]. Further research on polymorphisms associated with drought tolerance will aim to find combinations of SNP markers characteristic of individual regions of the common pine range.

## Figures and Tables

**Table 1:** Characteristics of the studied loci of *P. sylvestris*.

Locus	Primer Sequences (Forward/Reverse)	Gene Ontology Identificatory	Expected Product Size, bp
*Pinus-1*	AGTTCAAGGGTGGGTTGCAA/ GGTATGTGGTAGGATGGCCG	GO:0009788 negative regulation of abscisic acid-activated signaling pathway	601
*Pinus-2*	TTTGGGTGGCTGTCTGTGTT/ TCTGGTGCCAAAAACCCCAT	GO:0009788 negative regulation of abscisic acid-activated signaling pathway	587
*Pinus-3*	GCTGGAGCTGTTTGACACAA/ CACCGCACAAACAGTTCCAG	GO:0009415 response to water	592
*Pinus-4*	GTCACTCAGCAAGCGCAAAA/ CTTGCCACAGTCTTTGCCAC	GO:0071277 cellular response to calcium ion	590
*Pinus-5*	GGCAAAGGACAAGACCCAGA/ GATCCGTCGGCTCACATTCA	GO:0009415 response to water	565
*Pinus-6*	GTTCTGCTGCACTCTGGTCT/ GGAGCAGGTGCCTGAAGAAT	GO:0006952 defense response	587
*Pinus-7*	CCAGTCCGCGAATCCGATAA/ AACTCCGGCGTAAAGACTCC	GO:0009788 negative regulation of abscisic acid-activated signaling pathway	573
*Pinus-8*	TCTGTCGAAATGTGCACCGA/ TCATTGGCCTTCACTGACCC	GO:0009269 response to desiccation	518
*Pinus-9*	TCTATGAGCGATTCGGTGGC/ CAAAACCCGGGCTGAAACTG	GO:0009269 response to desiccation	533
*Pinus-10*	CCGCGGATAGTTATGCCCAT/ CATGCTCGATACCTGGCAGT	GO:0009788 negative regulation of abscisic acid-activated signaling pathway	579
*Pinus-11*	ATTCGACTTGCCCGGACTTT/ GAACGACAGTCTCAGGCCAA	GO:0009408 response to heat	504
*Pinus-12*	TCGCGAGTGAAGCTTCTGTT/ TTCCGGTGCATTGCTCTCTT	GO:0009408 response to heat	597
*Pinus-13*	TCGAGCGATGAAGAGGAGGA/ AATCACAACCCCACAACACG	GO:0009408 response to heat	502
*Pinus-14*	CCCTATCCTGGTTGCCGTTT/ AGCTCGCATTTACCTGTGCT	GO:0009788 negative regulation of abscisic acid-activated signaling pathway	575
*Pinus-15*	AGGCATTTGTGGTTTGGTGC/ CCTCCTTTTCTGGGCTCGTT	GO:0009408 response to heat	503
*Pinus-16*	TCGCACACAGAGAAGAGAGG/ TGGCAAATCATAACGCGCAG	GO:0009408 response to heat	574

**Table 2:** Primers and PCR conditions for amplification of *P. sylvestris* three loci.

Locus	Primer Sequences (Forward/Reverse)	PCR Conditions
*Pinus-11*	ATTCGACTTGCCCGGACTTT/ GAACGACAGTCTCAGGCCAA	Denaturation: 94 °C—5 min; 30 cycles: 94 °C—30 s, 60 °C—45 s, 72 °C—2 min, final elongation: 72 °C—10 min
*Pinus-12*	TCGCGAGTGAAGCTTCTGTT/ TTCCGGTGCATTGCTCTCTT	Denaturation: 94 °C—5 min; 30 cycles: 94 °C—30 s, 57 °C—45 s, 72 °C—2 min, final elongation: 72 °C—10 min
*Pinus-15*	AGGCATTTGTGGTTTGGTGC/ CCTCCTTTTCTGGGCTCGTT	Denaturation: 94 °C—5 min; 30 cycles: 94 °C—1 min, 55,7 °C—30 s, 72 °C—2 min, final elongation 72 °C—10 min

**Table 3:** Genes loci of *P. sylvestris* selected for nucleotide polymorphism study.

Locus	Product	Gene Ontology (GO)
*Pinus-11*	Small heat shock protein	GO:0009408 response to heat
*Pinus-12*	α-Crystallin domain of small heat shock protein	GO:0009408 response to heat
*Pinus-15*	MYB transcription factor	GO:0009408 response to heat

**Table 4:** The number of haplotypes and polymorphic sites revealed in the sequences of three *P. sylvestris* loci.

Locus/Population *		*Gn*	*Ch*	*Rm*	*Ar*	*Bl*	*Kr*	*Mh*	*Pr*	*Sl*	*Pl*	*Uk*	*Ln*	*Sk*	Total
*Pinus-11*	*h_n_*	4	4	4	6	6	5	6	3	4	4	4	3	4	19
	*S*	4	3	3	6	6	5	4	2	3	3	3	2	4	10
*Pinus-12*	*h_n_*	2	7	9	4	6	8	7	2	4	7	7	6	4	45
	*S*	1	9	36	3	6	18	10	1	5	9	16	8	5	84
*Pinus-15*	*h_n_*	4	3	2	3	2	0	2	3	3	3	3	5	2	6
	*S*	2	2	1	2	1	0	1	2	2	2	2	3	1	3

* *h_n_*—haplotype number; *S*—polymorphic site number per locus; maximal values of studied indices are highlighted in bold: *Gn*—the Gainskoe forest, *Ch*—Cherdynskoye, *Rm*—Bereznikovskoye, *Bl*—Tchaikovskoye, *Kr*—Sivinskoye, *Pr*—Kishertskoye, *Pl*—Dobryanskoye, *Uk*—Permskoye, *Ln*—Kudymkarskoye, *Sk*—Oktyabrskoye forestries of the Perm region; *Ar*—Kaslinskoye forest of the Chelyabinsk region; *Mh*—Duvanskoye, *Sl*—Salavatskoye forests of the Republic of Bashkortostan.

**Table 5:** Total haplotype and nucleotide diversity and neutrality test statistics for the three loci of *P. sylvestris*.

Locus *	*Hd*	*π*	*θ_W_*	*D_T_*
*Pinus-11*	0.737 (0.028)	0.004 (0.000)	0.006	−0.890
*Pinus-12*	0.630 (0.040)	0.005 (0.001)	0.032	−2.615
*Pinus-15*	0.620 (0.025)	0.003 (0.000)	0.002	0.925
Mean	0.662 (0.010)	0.004 (0.002)	0.013	-

* *Hd*—total haplotype diversity; π—nucleotide diversity; *θ_W_*—Watterson estimator of nucleotide diversity from the number of mutations; *D_T_*—Tajima *D*-test coefficient; standard deviations in brackets.

**Table 6:** Total haplotype and nucleotide diversity of *P. sylvestris* populations.

Indicators *	***PS_Ch***	***PS_Rm***	***PS_Kr***	***PS_Pl***	***PS_Pr***	***PS_Sk***	***PS_Sl***
*Hd*	0.661	0.675	0.533	0.653	0.478	0.589	0.592
*π*	0.004	0.003	0.007	0.004	0.004	0.002	0.002
*θ_W_*	0.003	0.003	0.009	0.006	0.004	0.001	0.002
Indicators *	***PS_Uk***	***PS_Gn***	***PS_Ln***	***PR_Ar***	***PS_Bl***	***PS_Mh***	***Total***
*Hd*	0.659	0.472	0.614	0.697	0.692	0.650	0.662
*π*	0.003	0.005	0.002	0.003	0.003	0.003	0.004
*θ_W_*	0.003	0.005	0.002	0.003	0.003	0.004	0.013

* *Hd*—total haplotype diversity; π—nucleotide diversity; *θ_W_*—Watterson estimator of nucleotide diversity from the number of mutations.

## Data Availability

The original contributions presented in the study are included in the article/Supplementary Material, further inquiries can be directed to the corresponding authors.

## Supplementary Materials

The following supporting information can be downloaded at: https://www.mdpi.com/article/10.3390/genes15101343/s1, Table S1. Studied populations of *P. sylvestris*. Figure S1. Electropherogram of amplification products of 16 primer pairs of Scots Pine. Size marker (M): DNA marker Step100 (Biolabmix, Russia), marked on the left in bp (100 bp to 1000 bp).
