# Supplementary material for: Polymorphic Loci of Adaptively Significant Genes Selection for Determining Nucleotide Polymorphism of Pinus sylvestris L. Populations in the Urals
Source: Genes (Basel). 2024 Oct 21;15(10):1343. doi: 10.3390/genes15101343 (PMC11507189; doi:10.3390/genes15101343)
Supplement: Supplementary file 1 [file genes-15-01343-s001.zip › genes-3257852-supplementary.pdf]

Supplementary Materials

Polymorphic Loci of Adaptively Significant Genes Selection for Determining Nucleotide Polymorphism of *Pinus sylvestris* L. Populations in the Urals

Nikita Chertov [1], Yana Sboeva [1], Yulia Nechaeva [1], Svetlana Boronnikova [1,*], Andrei Zhulanov [1], Victoria Pechenkina [1,2] and Ruslan Kalendar [3,4,*]

[1] Faculty of Biology, Perm State University, Bukireva, 15, 614990 Perm, Russia
[2] Perm Agricultural Research Institute—Branch of Perm Federal Research Center Ural Brunch Russian Academy of Sciences, 614532 Perm, Russia
[3] National Laboratory Astana, Nazarbayev University, Astana 010000, Kazakhstan
[4] Institute of Biotechnology HiLIFE, University of Helsinki, 00014 Helsinki, Finland
* Correspondence: svboronnikova@yandex.ru (S.B.); ruslan.kalendar@helsinki.fi (R.K.); Tel.: +358-294158869 (R.K.)

Supplementary Table S1. Studied populations of *P. sylvestris*

Populations ID	Location referencing	Coordinates	Height above sea level, m
PS_Ch	Cherdynsky forestry of Perm Krai	N: 60°25'48<br>E: 56°18'36	189
PS_Gn	Gainsky forestry of Perm Krai	N: 60°20'24<br>E: 53°50'24	148
PS_Rm	Bereznikovsky forestry of Perm Krai	N: 59°08'24<br>E: 56°47'24	123
PS_Ln	Kudymkarsky forestry of Perm Krai	N: 58°41'24<br>E: 54°41'24	201
PS_Pl	Dobryansky forestry of Perm Krai	N: 58°18'36<br>E: 56°16'12	172
PS_Kr	Sivinsky forestry of Perm Krai	N: 58°07'48<br>E: 54°10'12	183
PS_Bl	Tchaikovsky forestry of Perm Krai	N: 57°42'36<br>E: 55°16'12	132
PS_Uk	Permsky forestry of Perm Krai	N: 57°39'00<br>E: 55°25'12	116
PS_Pr	Kishertsky forestry of Perm Krai	N: 57°21'36<br>E: 57°10'12	171
PS_Sk	Oktyabrsky forestry of Perm Krai	N: 57°07'48<br>E: 57°22'48	232
PS_Ar	Kaslinsky forestry of Chelyabinsk region	N: 55°59'24<br>E: 60°30'00	375
PS_Mh	Duvansky forestry of Republic of Bashkortostan	N: 55°58'48<br>E: 58°17'24	287
PS_Sl	Salavatsky forestry of Republic of Bashkortostan	N: 55°10'48<br>E: 58°10'48	328

N - north latitude, E - east longitude.

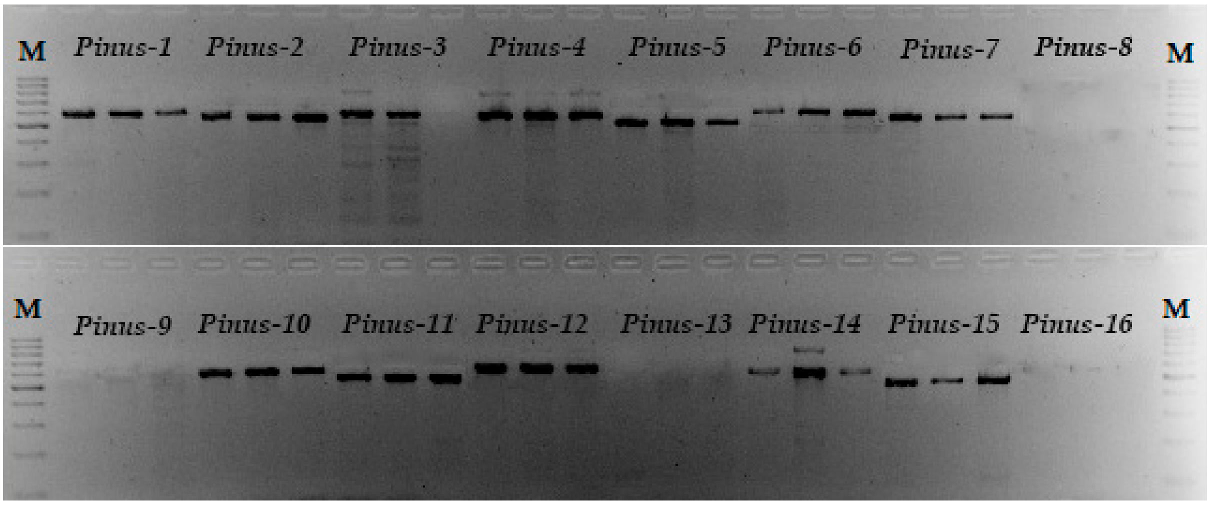

Supplementary Figure S1. Electrophoregram of amplification products of 16 primer pairs of Scots pine. Size marker (M): DNA marker Step100 (Biolabmix, Russia), marked on the left in bp (100 bp to 1000 bp).
